# The Participatory Zeitgeist in Health Care: It is Time for a Science of Participation

**DOI:** 10.2196/15101

**Published:** 2020-01-10

**Authors:** Victoria Jane Palmer

**Affiliations:** 1 The Department of General Practice Melbourne Medical School The University of Melbourne Melbourne Australia

**Keywords:** participation, participatory methods, health care improvement, quality improvement, coproduction, co-design, theory of science

## Abstract

Participation in health care is currently the zeitgeist/spirit of our times. A myriad of practices characterizes this “participatory Zeitgeist” in contemporary health care, which range from patients and professionals collaborating as partners in service delivery and treatment decision-making, to crowdsourced cures and participation in online communities, to using health apps, to involvement in health care quality improvement initiatives for systems redesign using coproduction and co-design methods. To date, patient engagement and participation in online communities and the use of apps have received a good deal of attention in participatory medicine. However, there has been a less critical examination of participation in health care planning, design, delivery, and improvement. In the face of what Thomas Kuhn called a scientific revolution, we are presented with the opportunity to re-examine some of the assumptions underpinning participation in health care and some of the emerging anomalies and weaknesses in the current science. This re-examination will allow the development of a new paradigm, a science of participation. In this science, we can systematically test, refine, and advance participation in health care to build a unifying language and theories from across the interdisciplinary fields of participatory design, medicine, and research to develop and test models to explain impacts and outcomes. A science of participation will allow the emergent and unexplained facts to be addressed in the current participatory mood of health care planning, design, delivery, and improvement.

## Introduction

Contemporary health care planning, design, delivery, and improvement is characterized by “a participatory Zeitgeist” [1], where participation is enacted within intellectual, social, political, cultural, and moral pursuits that are reflective of and shaped by a participatory spirit of the times, mood of the times, or spirit of the age [2]. There is no doubt that broader socio-cultural trends toward participation in health care intersect with this participatory Zeitgeist [3]. These trends include the involvement of the public in data collection for health research, initiatives in patient-led and crowdsourced research [4], the use of health care apps for self-management, greater emphasis on users in design phases, and embedding lived-experience within research and health care policy formulation. The participatory spirit also includes the drive for experience to be considered an equal source of evidence, as shown by the experts-by-experience and the engaged, empowered, and emancipated patient (e-patient) movements [5]. Alongside the e-patient movement is the enabled health care professional who is ideally supported by an elegant health care system designed to foster “unhurried and kind care” [6].

These shifts in participation are coupled with increased involvement in health systems planning, design, and quality improvement in unprecedented ways via participatory methods such as coproduction (including the variants of co-design, coinnovation, and cocreation) [7]. In this regard, participation has itself become a critical agent in health care planning, redesign, delivery, quality improvement, and systems transformation [8]. As the economist Elinor Ostrom noted, participation creates a synergistic value through the active roles people have in producing public goods and health care services that are of consequence to them [9]. While synergistic value is essential for recognition that people are coproducers of public goods, such as health care and associated services, participation in the “design and implementation of new policies, systems and services as well as patient care and clinical decision-making” [4] is now so prolific that it is time to genuinely consider the need for a science of participation in health care.

### Why Do We Need a Science of Participation?

As a term, science refers to the systematic study, organization, and synthesis of knowledge of phenomenon and the mobilization of theories, concepts, and methods to better understand the what, why, and how that phenomenon works [10]. Calling for a science does not mean that existing theories and concepts are not available or relevant to building a systematic evidence base, or to synthesizing knowledge; indeed, there are long-standing traditions in the participatory paradigms [11]. Instead, the call for a science of participation suggests that there are currently three critical gaps that exist in the examination and interpretation of the phenomenon of participation. These gaps relate to:

- The need for a unifying language to bring together the many and varied ways that participation occurs in health care design, delivery, and improvement;
- The need to develop and apply explanatory theories and models to better understand how participation occurs and what is produced. This includes attending to different participatory roles of people, such as patients, the family/carers, clinicians/providers, designers, researchers, or government representatives, and;
- The need to generate a systematic evidence base of impact and outcome using theories, models, and measures developed by the participatory fields.

A science of participation will, by nature, be interdisciplinary, and it will intersect with paradigms across participatory design, participatory medicine, participatory research methods, and across approaches for engagement, collaborative decision-making, and change. A science of participation will mobilize existing knowledge, theories, and frameworks with a focus on unification, not replication, and synthesis, not reinvention. It will allow the identification of value creation in terms of impacts and outcomes from within the field. The following parts of this viewpoint will outline how a science of participation can contribute to addressing the three critical gaps of the phenomenon of participation.

### Gap 1: The Need for a Unifying Language

A core rationale for a science of participation is that we are amid a scientific revolution in the participatory paradigm. Kuhn described the scientific revolution as a process by which normal science continues while there is a consensus about a framework, at least until anomalies emerge. Here we can use two examples to illustrate this point about anomalies. In the first case, coproduction and co-design frameworks in health care quality improvement have continued to be used as normative quality improvement methods. However, anomalies and facts that are difficult to explain in the context of the current paradigm have started to emerge and generate weaknesses. For example, cracks are emerging in the increased calls for evidence of impact and outcome from coproduction and co-design. Now, various authors suggest that it is the outcomes of coproduction and not the processes that achieve those outcomes that should be measured [12]. Coupled with this is a growing concern that the terms coproduction and co-design are losing meaning and creating weaknesses in the standard science too because they are being overused without attendance to the values, principles, and practices that ought to underpin them [13-15]. Indeed, there is variability in how coproduction and co-design are defined, so determining the different effects, impacts, and outcomes of various approaches is a challenge that will require an agreed upon vocabulary [14].

The second case for an emergent anomaly in the current science is illustrated in a recent article by DeBronkart on patient engagement [16]. In this paper, DeBronkart described how medicine has an outdated paradigm of the patient as a passive recipient, which has created weakness and the possibility for a new paradigm, that of the e-patient. This e-patient is a responsible driver of health, who shares part of the work as appropriate to their role and abilities [16]. Thus, in Kuhn’s revolution, weaknesses in science provide the opportunity for a paradigm shift where underlying assumptions are re-examined, and a potentially new paradigm emerges [17]. This new paradigm in health care design, delivery, and improvement is a science of participation.

### Gap 2: The Need to Develop Explanatory Theories and Models of Change

To date, participation in health care planning, design, delivery, and improvement has been primarily explained and examined through existing paradigms of implementation science, improvement science, and citizen science. While these are important sciences from which we can learn, they do not provide the field with the explanatory theories and models needed to re-examine the participation paradigm in conjunction with the anomalies and weaknesses outlined above, or concerning the phenomena of participation that is occurring in health care. That is, a science of participation is needed to identify the impacts and outcomes we ought to expect of coproduction and co-design. Moreover, it is needed to identify if participation (according to particular methods and approaches) in design, delivery, and improvement results in better patient experiences, quality care, and improved health outcomes. This includes understanding and evaluating the role of health care professionals in the participatory Zeitgeist.

To address these complexities, models and theories that have explanatory force for the phenomena of participation are required. In Table 1, the three currently existing and dominant paradigms used to describe participation in health care design, delivery, and improvement are briefly outlined [18-20]. Each of these paradigms has established traditions that are not entirely covered in their brief descriptions; however, the aim is to highlight the gaps in these sciences for attending specifically to participation. It is also acknowledged that there are several intersecting traditions across these sciences (eg, participatory design or distributed thinking and participatory medicine itself) that have influenced their development, which has not been covered in this summary.

In our re-examination of the assumptions that underpin participation in health care, there is an opportunity to synthesize what is a largely fragmented and inconclusive evidence base [19] and apply explanatory theories developed from our field. Existing work in participatory design can assist. Steen, for example, articulated the importance of virtue ethics in participatory design practice [21]. He outlined the essential virtues of cooperation, curiosity, creativity, empowerment, and reflexivity for designers and noted, drawing on MacIntyre’s work in ethics, that virtues are not only about a disposition to “act…but also to feel in particular ways” [21]. More recently, an explanatory theoretical model of change identified eight mechanisms seen to be critical for facilitation of change in co-design and coproduction in health care improvement: recognition, dialogue, cooperation, accountability, mobilization, creativity, enactment, and attainment [2]. The explanatory theoretical model positioned these mechanisms within the relational contexts of co-design and coproduction activities and described some ideal transitions that might be expected in these activities. These included moving from being isolated (I), to somewhat recognizing experiences might be shared (I to Them), to sharing experiences and developing understanding (Them to You), to embracing a collective sense of change (You to Us), to all working together to achieve that change (Us to We) [2]. Such theoretical models are essential for building the conditions for participation and to interpret the impacts and outcomes.

**Table 1:** Distinction between citizen, implementation, and improvement sciences

	Citizen Science [15]	Implementation Science [16]	Improvement Science [17]
Historical tradition	Natural Sciences, such as bird observations, classifications, and collection of data by “non-scientists” for use by scientists. Participants as volunteer data collectors with aim to collect large datasets. Variants on this term are used in the literature and include civic science, community environmental policing, street science, popular epidemiology, and crowd science.	The implementation of evidence into practice and translation gap. Identification of evidence into practice roadblocks to improve implementation.	The quality chasm and improvement of quality of care to increase safety, with a focus on changing physician behavior. Highly influenced by the United States Institute of Medicine Quality Chasm reports.
Original purpose	To address some of the problems of time, space, and large amounts of data required for the biological sciences. People being able to collect data in different geographical locations. Some work was undertaken in medical research, such as Malaria Spot.	To promote uptake of evidence-based interventions into practice and policy. Early work had empirical focus with less attention to theory.	Systems-level work to improve the quality, safety, and value of health care. Premised on the idea that improvement would result in greater efficiencies in terms of both patient outcomes and cost.
Contemporary variants	A science that is focused on the needs and concerns of citizens and is developed and enacted by citizens. Shift from the person as the object of study to the citizen as a research subject (for data collection and analysis). Part of the evolution of digital humanities where large repositories of data can be collected (eg, Zooniverse platform). Also used in human-computer interaction studies to develop gamified solutions from data people contribute.	Progression of theoretical models and approaches to better understand and explain how and why implementation fails or succeeds. Identification of the conditions for implementation readiness in different settings.	Greater focus on the association between patient experience of care and quality, safety, and value of health care. Embedding public and patient in the processes of identification of systems of change areas, design, and co-development of solutions with professionals. Working in a partnership model between academia and frontline clinicians. Contribution to theories of how change happens.

### Gap 3: A Systematically Generated Evidence Base of Impact and Outcomes

The call for a science of participation is coupled with the need for systematic examination and observation of impact and outcome. There has been a growth in literature outlining an expectation that we should see evidence of impact from coproduction [22-26], and there is an expectation that participation from patients, carers/families, and service users increases patient-centered outcomes, improves professional morale, and increases health and well-being; however, the measurement of this has been inconsistent and almost absent. To date, one cluster randomized controlled trial has been conducted to test the assumption that a participatory, co-design, quality improvement method may improve individual, psychosocial, recovery outcomes: the CORE Study (2013-2017) [23]. Some evidence indicates that collective coproduction reduces diagnostic error in hospitals [24], and survey results from the United Kingdom and from Australian and European nations have shown that a turn to participation via coproduction is more likely when government shortfalls in performance prevail [25].

When Don Berwick called for a science of improvement for health care over ten years ago, he highlighted that disputes for the development of a science were more likely to be about epistemological disagreement rather than the type of research required to generate an evidence base [27]. A distinguishing feature of the current participatory times is the increased recognition of the importance of lived-experience (experiential knowledge) and patient-led change [11]. This has traditionally raised an epistemological tension between advocates for participatory paradigms and evidence-based paradigms. It is time to cross the epistemological bridges and establish a science of participation that helps to explain impacts, document outcomes, and bring theories together into a unifying whole.

Almost 25 years ago, Ostrom also concluded that “contrived walls separating the analysis of potentially synergetic phenomena into separate parts misses the potential for synergy” [10]. The current state of play in participation in health care offers good ground for synergies among diverse theoretical and practical approaches from participatory design, participatory medicine, participatory action research, co-design and coproduction, to patient engagement, the e-patient movement, and enabled health care professionals. The next steps involve our building of a science of participation that contributes to the identification of the components and features of an elegant [6] health system to support participation. These steps include but are not limited to: (1) knowledge synthesis of the current phenomena of participation in health care design, delivery, and improvement to organize our somewhat disparate and divergent strands of fragmented evidence; (2) systematic study of participation to identify impacts and outcomes; and (3) harnessing existing theories, concepts, and methods to explain and interpret phenomena so that we might develop new models based on our science as appropriate. Now is the time for a science of participation.

## Abbreviations

e-patient: engaged, empowered, and emancipated patient
